# Computational staining of CD3/CD20 positive lymphocytes in human tissues with experimental confirmation in a genetically engineered mouse model

**DOI:** 10.3389/fimmu.2024.1451261

**Published:** 2024-10-28

**Authors:** Xiang Li, Casey C. Heirman, Ashlyn G. Rickard, Gina Sotolongo, Rico Castillo, Temitayo Adanlawo, Jeffery I. Everitt, Jeffery B. Hodgin, Tammara L. Watts, Andrew Janowczyk, Yvonne M. Mowery, Laura Barisoni, Kyle J. Lafata

**Affiliations:** ^1^ Department of Electrical and Computer Engineering, Duke University, Durham, NC, United States; ^2^ Medical Physics Graduate Program, Duke University, Durham, NC, United States; ^3^ Department of Radiation Oncology, Duke University, Durham, NC, United States; ^4^ Department of Pathology, Duke University, Durham, NC, United States; ^5^ Department of Pathology, University of Michigan, Ann Arbor, MI, United States; ^6^ Department of Head and Neck Surgery & Communication Sciences, Duke University, Durham, NC, United States; ^7^ Department of Biomedical Engineering, Emory University and Georgia Institute of Technology, Atlanta, GA, United States; ^8^ Department of Oncology, Division of Precision Oncology, Geneva University Hospitals, Geneva, Switzerland; ^9^ Department of Diagnostics, Division of Clinical Pathology, Geneva University Hospitals, Geneva, Switzerland; ^10^ Department of Radiation Oncology, UPMC Hillman Cancer Center/University of Pittsburgh, Pittsburgh, PA, United States; ^11^ Department of Medicine, Division of Nephrology, Duke University, Durham, NC, United States; ^12^ Department of Radiology, Duke University, Durham, NC, United States

**Keywords:** *Rag2* knockout (KO) mouse, inflammatory response, lymphocytes, digital pathology, pathomics, deep learning, experimental validation

## Abstract

**Introduction:**

Immune dysregulation plays a major role in cancer progression. The quantification of lymphocytic spatial inflammation may enable spatial system biology, improve understanding of therapeutic resistance, and contribute to prognostic imaging biomarkers.

**Methods:**

In this paper, we propose a knowledge-guided deep learning framework to measure the lymphocytic spatial architecture on human H&E tissue, where the fidelity of training labels is maximized through single-cell resolution image registration of H&E to IHC. We demonstrate that such an approach enables pixel-perfect ground-truth labeling of lymphocytes on H&E as measured by IHC. We then experimentally validate our technique in a genetically engineered, immune-compromised *Rag2* mouse model, where *Rag2* knockout mice lacking mature lymphocytes are used as a negative experimental control. Such experimental validation moves beyond the classical statistical testing of deep learning models and demonstrates feasibility of more rigorous validation strategies that integrate computational science and basic science.

**Results:**

Using our developed approach, we automatically annotated more than 111,000 human nuclei (45,611 CD3/CD20 positive lymphocytes) on H&E images to develop our model, which achieved an AUC of 0.78 and 0.71 on internal hold-out testing data and external testing on an independent dataset, respectively. As a measure of the global spatial architecture of the lymphocytic microenvironment, the average structural similarity between predicted lymphocytic density maps and ground truth lymphocytic density maps was 0.86 ± 0.06 on testing data. On experimental mouse model validation, we measured a lymphocytic density of 96.5 ± %1% in a *Rag2^+/-^* control mouse, compared to an average of 16.2 ± %5% in *Rag2^-/-^* immune knockout mice (p<0.0001, ANOVA-test).

**Discussion:**

These results demonstrate that CD3/CD20 positive lymphocytes can be accurately detected and characterized on H&E by deep learning and generalized across species. Collectively, these data suggest that our understanding of complex biological systems may benefit from computationally-derived spatial analysis, as well as integration of computational science and basic science.

## 1 Introduction

Inflammatory mechanisms and a well-regulated immune response are essential for protecting against pathogens, preventing chronic inflammatory conditions, and responding to tissue damage (1). In contrast, immune dysregulation can lead to a variety of health issues, including autoimmune diseases, chronic inflammation, susceptibility to infection, and the development and progression of cancer. Inflammatory processes mediated by B and T lymphocytes play a major role in both cancer immunity (e.g., immune surveillance, tumor infiltrating lymphocytes, stromal inflammation) and therapy (e.g., immune checkpoint inhibitors, CAR T-cell therapy, etc.). Precision and accuracy in quantifying lymphocytic inflammation are critical for diagnosis and classification of certain disease processes, enabling use of lymphocytic infiltration as a prognostic marker, and potentially developing targeted treatments that improve patient outcomes. Pathomics, i.e., high-throughput extraction and analysis of features from digital pathology images, represents a promising approach to derive a multi-scale mathematical representation of lymphocytic infiltration phenotypes for both clinical and research purposes.

Image-based quantification and characterization of lymphocytes on digitized tissue biopsies via deep learning enables spatial interrogation of the tumor immune microenvironment, thus providing a better description of the inflammatory spatial phenotype than other techniques, such as flow cytometry or RNA sequencing. For example, deep learning has been applied to count CD3 positive T lymphocytes stained by immunohistochemistry (IHC) on whole slide images (WSIs) and/or to quantify CD8 expression in prostate (2), colon (2, 3), neuroblastoma (4), gastric (5), breast (6), and lung (7) cancers. Deep learning has also been used to quantify other relevant inflammation-related protein markers on IHC such as Inducible T-cell COStimulator (ICOS), which is involved in T-cell activation and adaptive immune response (8).

Notably, IHC is expensive and often not feasible in routine pathology and exploratory retrospective research. Consequently, there is a growing need to develop deep learning algorithms that can accurately detect lymphocytes on WSIs from hematoxylin and eosin (H&E) stained tissue (i.e., the standard staining technique used to visualize tissue morphology and structure). Deep learning-derived digital staining (9) of H&E WSIs can facilitate efficient and unbiased detection and quantification of different cell types across various tissues and diseases, such as melanoma (10), breast cancer (11–13), colorectal cancer (13), and testicular cancer (14).

However, the application of deep learning to characterize the immune response on digital pathology presents several key challenges. First, a notable constraint is that these models generally require manual annotation of class labels on H&E during the training process. Obtaining accurate annotations for large numbers of lymphocytes is a labor-intensive, time-consuming process that is limited by both inter- and intra-observer variability. Second, while these models excel at pattern recognition, they lack the mechanistic understanding required to rigorously evaluate immune responses. That is, deep learning may not fully capture basic biological characteristics beyond surface-level image representation. This hinders the ability of these models to provide deeper insight into the underlying mechanisms of different immune responses, highlighting the need for complementary testing of deep learning solutions under controlled experimental conditions.

**Figure 1:**
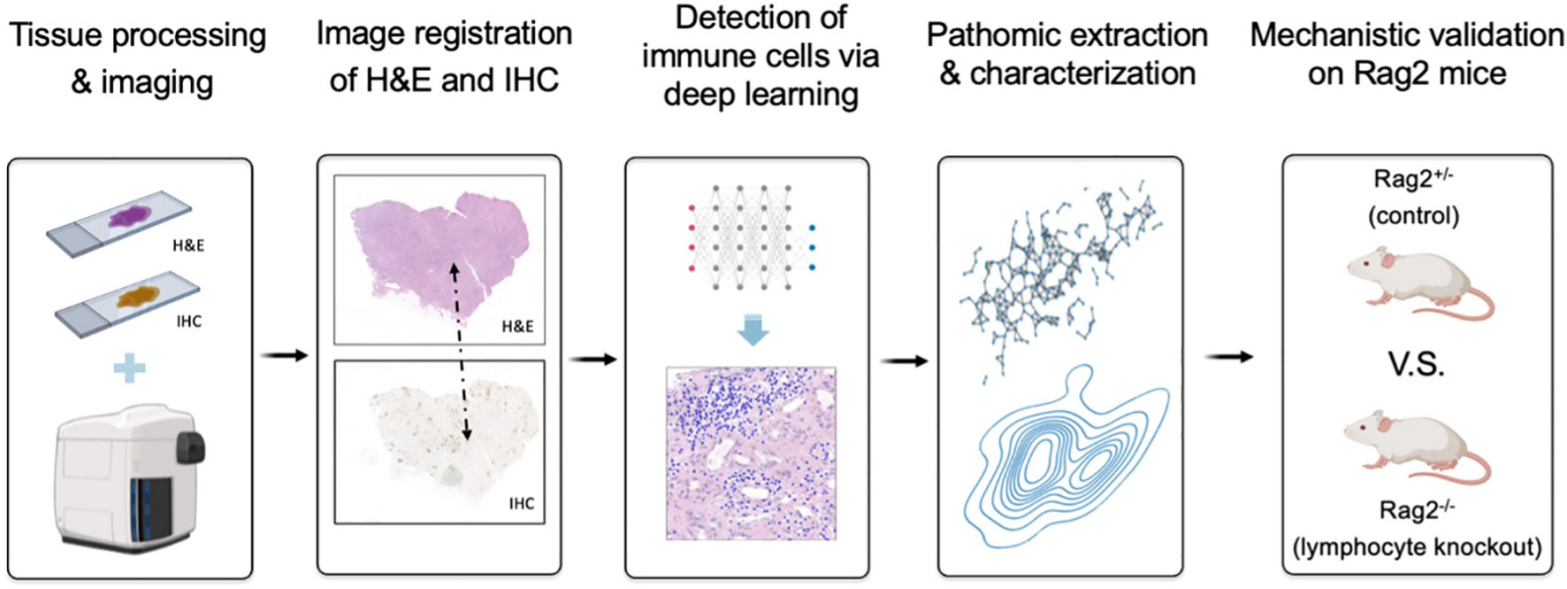
Overview of research design for lymphocyte deep learning model development and evaluation. We first developed a knowledge-guided deep learning framework to measure lymphocytes on H&E-stained kidney tissue, where the fidelity of training labels was maximized through single-cell resolution image registration of H&E to IHC staining with a cocktail of anti-CD3 and anti-CD20 antibodies to stain T and B lymphocytes, respectively. Following traditional statistical testing of the model based on receiver operating characteristic (ROC) curve analysis, we then characterized pattern-preserving features of the immune microenvironment based on pathomic feature extraction and calculated their error rates relative to the shift-invariant IHC antibody measurements. Finally, we performed a preclinical experiment to confirm that our deep learning model is able to identify lymphocytes in a genetically engineered mouse model, where *Rag2* knockout mice without mature lymphocytes are used as a negative control.

In this paper, we address these challenges via an integrated research design that combines computational (i.e., “dry lab”) techniques and experimental (i.e., “wet lab”) rigor (**Figure 1**). First, we developed a knowledge-guided deep learning framework to measure lymphocytes on H&E tissue, where the fidelity of training labels is maximized through single-cell resolution image registration of H&E to IHC. We demonstrate that such an approach enables pixel-perfect ground-truth labeling of lymphocytes on H&E as measured by IHC. Second, we move beyond conventional statistical testing of our deep learning model by subjecting it to rigorous testing in a genetically engineered mouse model, where *Rag2* knockout mice lacking mature lymphocytes are used as a negative experimental control. Our results demonstrate that the immune microenvironment can be accurately captured on H&E by deep learning across species and tissue types, generalizing a model developed on kidney tissue from humans to various human cancers, as well as splenic and thymic tissues form mice. Collectively, these data suggest that our understanding of complex biological systems may benefit from combining data-driven insights with empirical biology.

## 2 Methods

### 2.1 Experimental tissue preparation and whole slide image acquisition

**Figure 2:**
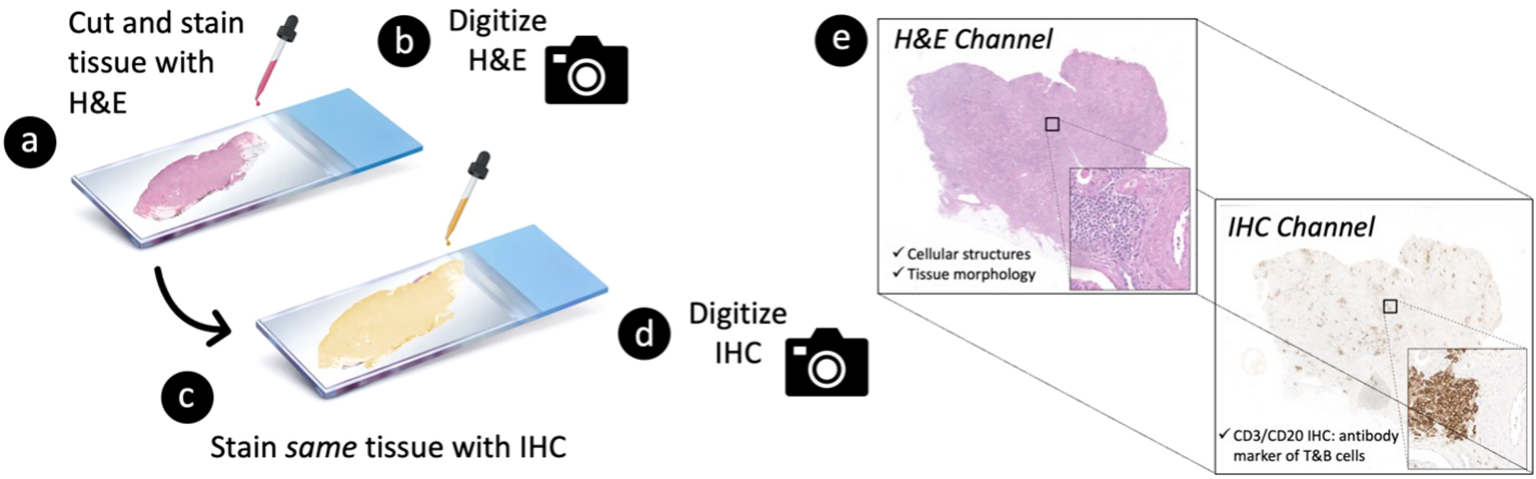
Tissue processing strategy to generate 2-channel, multi-contrast digital images of paired hematoxylin and eosin (H&E) vs. immunohistochemistry (IHC) stains at single-cell resolution. A 2-micron thick tissue specimen is first **(A)** stained with H&E and subsequently **(B)** digitized into a whole slide image (WSI) at 40X magnification. The specimen is then **(C)** re-stained with CD3/CD20 IHC (i.e., antibody markers of T and B cells, respectively) and **(D)** re-imaged on the same whole slide scanner. This process results in **(E)** a 2-channel, multi-contrast WSI encoding both (i) the morphological characteristics of the tissue on H&E and (ii) the corresponding CD3/CD20 expression profile of the tissue on IHC.

Formalin-fixed, paraffin-embedded tissue blocks from nephrectomies of patients (N=18) with kidney cancer and moderate-to-severe inflammation in renal tissue away from the cancer were obtained from the Duke University Pathology Paraffin Tissue Archives. Nephrectomies were chosen as the basis of generating scalable deep learning training examples because they often involve significant inflammation, thus providing a rich source of lymphocytes and diverse inflammatory microenvironments. Tissues were cut at 2 microns thick, stained with H&E (**Figure 2A**), and digitized into WSIs at 40X magnification (0.25 µm/pixel) using a Leica Aperio AT2 whole slide scanner (**Figure 2B**). Following H&E image acquisition, the same tissue specimens were re-stained with a CD3/CD20 IHC cocktail to detect T (CD3+) lymphocytes and B (CD20+) lymphocytes (**Figure 2C**). The IHC-stained specimens were then re-scanned at 40X magnification on the same Leica Aperio AT2 imaging system (**Figure 2D**), resulting in 2-channel, multi-contrast WSIs encoding both tissue morphology (on H&E) and tissue CD3/CD20 expression (on IHC) (**Figure 2E**).

### 2.2 Image registration of H&E and IHC at single-cell resolution

**Figure 3:**
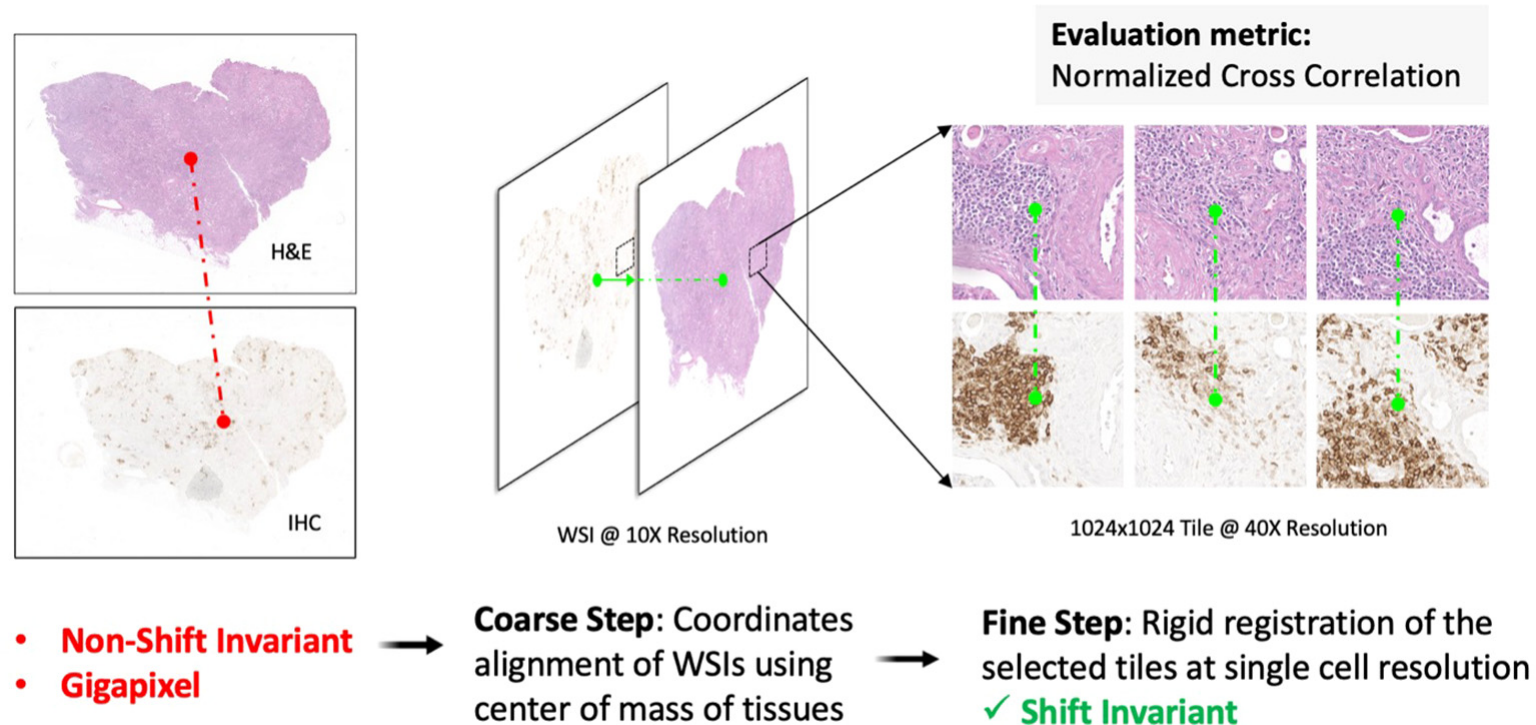
Multiscale H&E to IHC image registration. First, a coarse registration step provides an initial alignment of the entire tissue based on the center-of-mass shift between the H&E image and the IHC image. Second, a fine registration step aligns individual nuclei on tiled sections (1024x1024 pixels) of tissue, resulting in a one-to-one matching of nuclei across the H&E channel and the IHC channel. The 2-channel, shift-invariant tiled image data is stored for downstream deep learning model development.

To minimize shift artifacts, each H&E image was co-registered to its matched IHC image to map the corresponding location of CD3/CD20 antibody expression on IHC to individual nuclei on H&E. A multiscale, two-step rigid registration process (**Figure 3**) was implemented as follows. First, the H&E and IHC images were each downsampled to 25% of their native resolution to accommodate RAM constraints, and their center-of-mass (CoM) was calculated as a coarse tissue landmark. The IHC image was shifted to the H&E image based on a CoM alignment. Second, 1024x1024 tiles of pixels were stochastically sampled at full resolution from both H&E and IHC images at regions of high CD3/CD20 signal. The IHC tiles and H&E tiles were co-registered at single-cell resolution and stored as a tiled database for downstream deep learning model development. The accuracy of image registration was quantified based on Normalized Cross Correlation (NCC).

### 2.3 Knowledge-based lymphocyte labeling to generate deep learning training data

**Figure 4:**
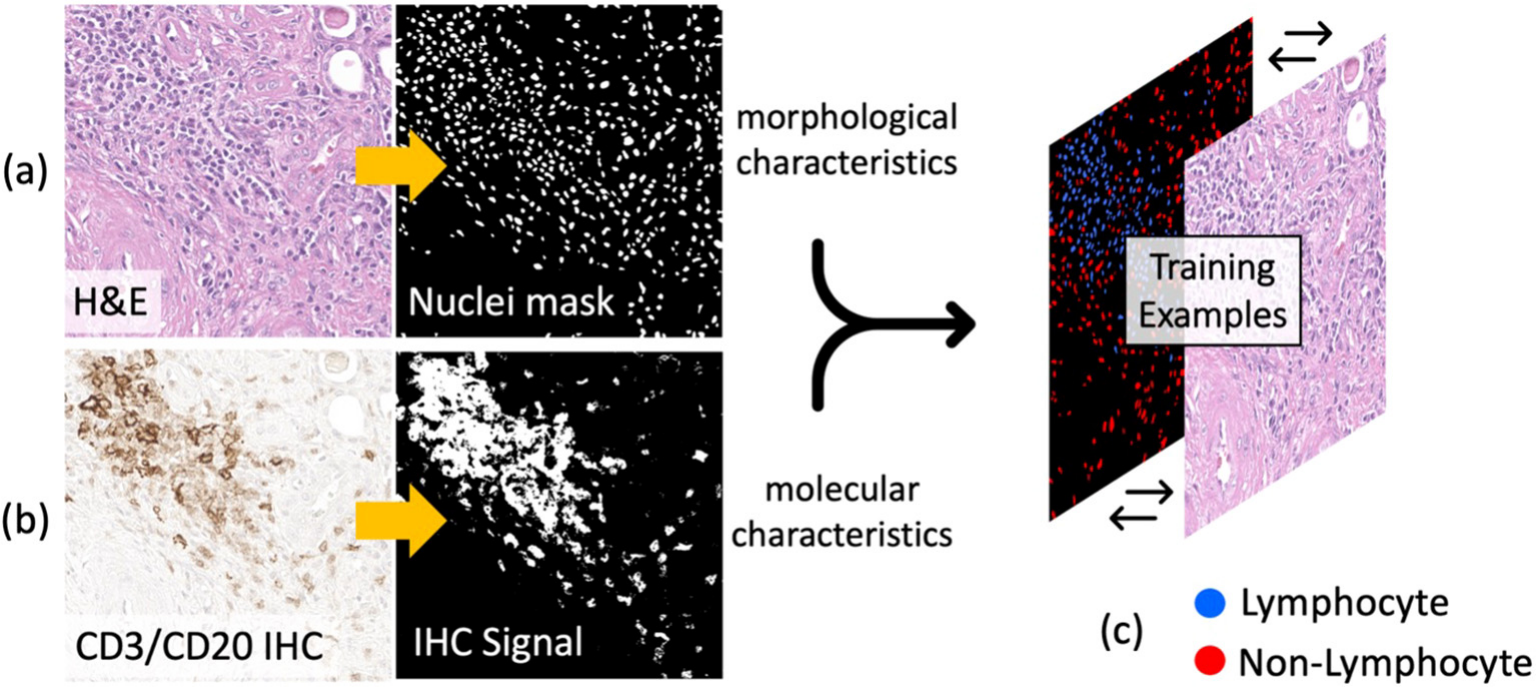
Automatic lymphocyte labeling to generate deep learning training data at single-cell resolution. **(A)** Nuclei are first identified and segmented using a pretrained StarDist model. **(B)** The CD3/CD20 IHC signal (brown stain) is then thresholded and mapped onto the segmented nuclei to identify lymphocytes on H&E. **(C)** Deep learning training examples are curated by labeling nuclei as either lymphocytes (positive class: overlap between nuclear contour and IHC signal) or non-lymphocytes (negative class: non-overlap between nuclear contour and IHC signal).

The set of shift-invariant, co-registered H&E/IHC tile pairs (**Figure 3**) were used to curate single-cell deep learning training data labels. First, using a pre-trained StartDist deep learning model (15), all nuclei (irrespective of cell type) were detected and segmented on each H&E image tile (**Figure 4A**). Next, color deconvolution of the CD3/CD20 stain was used to isolate and threshold lymphocyte-positive regions on each IHC image tile (**Figure 4B**). Finally, nuclei on H&E with at least 70% of their pixels overlapping with the corresponding IHC signal were labeled as lymphocytes and denoted as the positive class label (**Figure 4C**). This 70% overlap threshold was empirically determined to balance precision and accuracy, as requiring 100% overlap was too restrictive given that IHC stains can leak beyond the nuclei, causing variability and potential errors. The nuclei on H&E that were non-overlapping with IHC were labeled as non-lymphocytes and denoted as the negative class label (**Figure 4C**).

### 2.4 Deep learning detection of lymphocytes on H&E based on prior-knowledge of IHC

**Figure 5:**
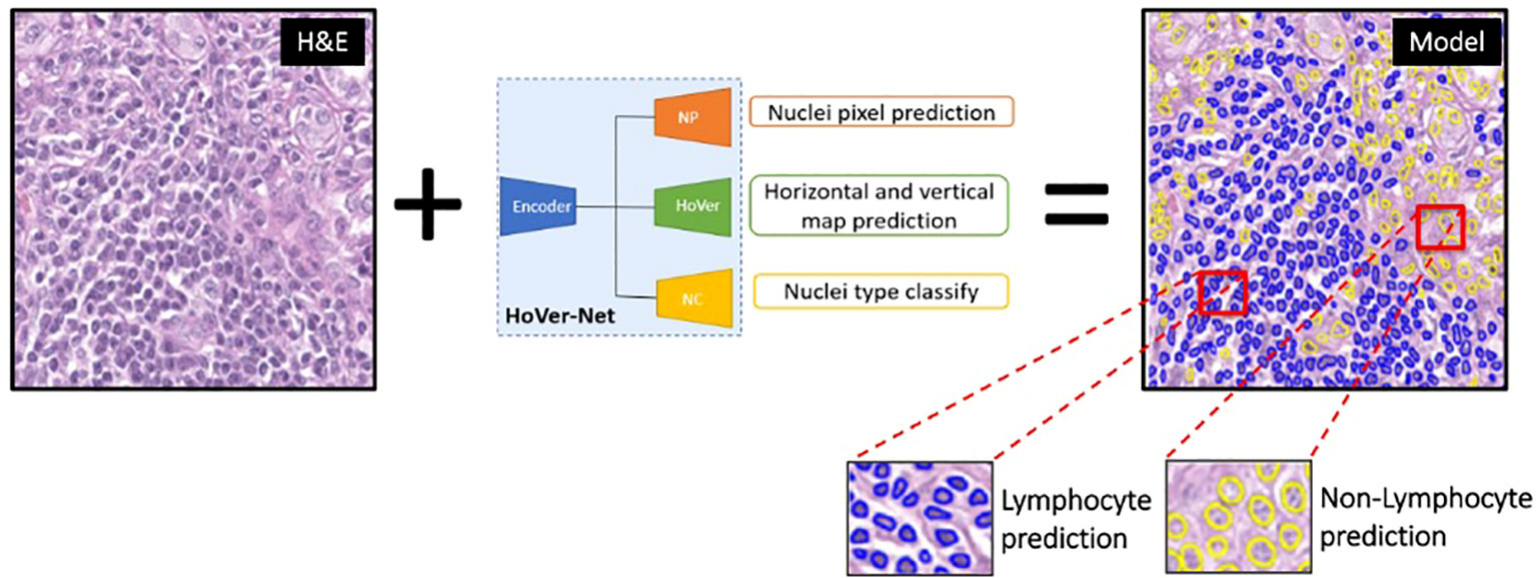
Hover-Net model architecture for lymphocyte identification. This multi-task deep learning framework was implemented to simultaneously detect, segment, and classify lymphocytes (blue predictions) and non-lymphocytes (yellow predictions) on H&E images.

Using the IHC-guided nuclear labels (**Figure 4**), a deep learning model was developed to detect lymphocytes on H&E in the absence of IHC (**Figure 5**). Our model was based on the HoverNet architecture (16), which is a multi-task deep learning framework to simultaneously detect, segment, and classify nuclei. The HoverNet architecture consists of one image encoding branch and three decoding branches, each with a specific task: (1) a nuclear segmentation branch, which is responsible for segmentation of nuclei; (2) a nuclear classification branch, which is responsible for classifying the segmented nuclei into types; and (3) an instance separation branch, which is responsible for separating overlapping nuclei based on distance vector fields (e.g., hover maps) from each pixel inside a nucleus to its center. By encoding directional information, this state-of-the-art architecture is designed to effectively handle the challenges of nuclear segmentation in complex tissue environments with dense regions of overlapping nuclei, such as in lymphocytic inflammation.

The data were split at the patient level into a training/validation cohort (N=280 tiles from N=14 WSIs) and an internal testing cohort (N=40 tiles from N=4 WSIs). The deep learning input patch size was 270x270 pixels, which was randomly extracted 50 times from each 1024x1024 tile. During training, concurrent parameter optimization was based on Dice loss for nuclear segmentation, binary cross entropy for nuclear classification, and mean square error loss for distance prediction between each nuclear pixel and its center. Training utilized the Adam optimizer (100 epochs) with learning rate linear degradation every 30 epochs. Various data augmentation techniques were applied to increase the variability of the training data. Model parameters were fine-tuned during training with an 8:2 ratio of training and validation, and independently evaluated on the held out internal testing cohort. Specific hyperparameters are provided in **Table 1**.

**Table 1:** Hyperparameter settings for Hover-Net.

Hyperparameters	Settings
Learning Rate	1e-3 with linear decay every 30 epochs
Data Augmentation	Affine, Flip, Crop, Blur, Color, Contrast
Optimizer	Adam
Epoch	100
Loss Function	BCE loss + Dice loss + MSE loss
Batch Size	16
Pretrained Weights	ImageNet-ResNet50-Preact weights

### 2.5 Lymphocyte deep learning model evaluation and multi-scale performance metrics

**Figure 6:**
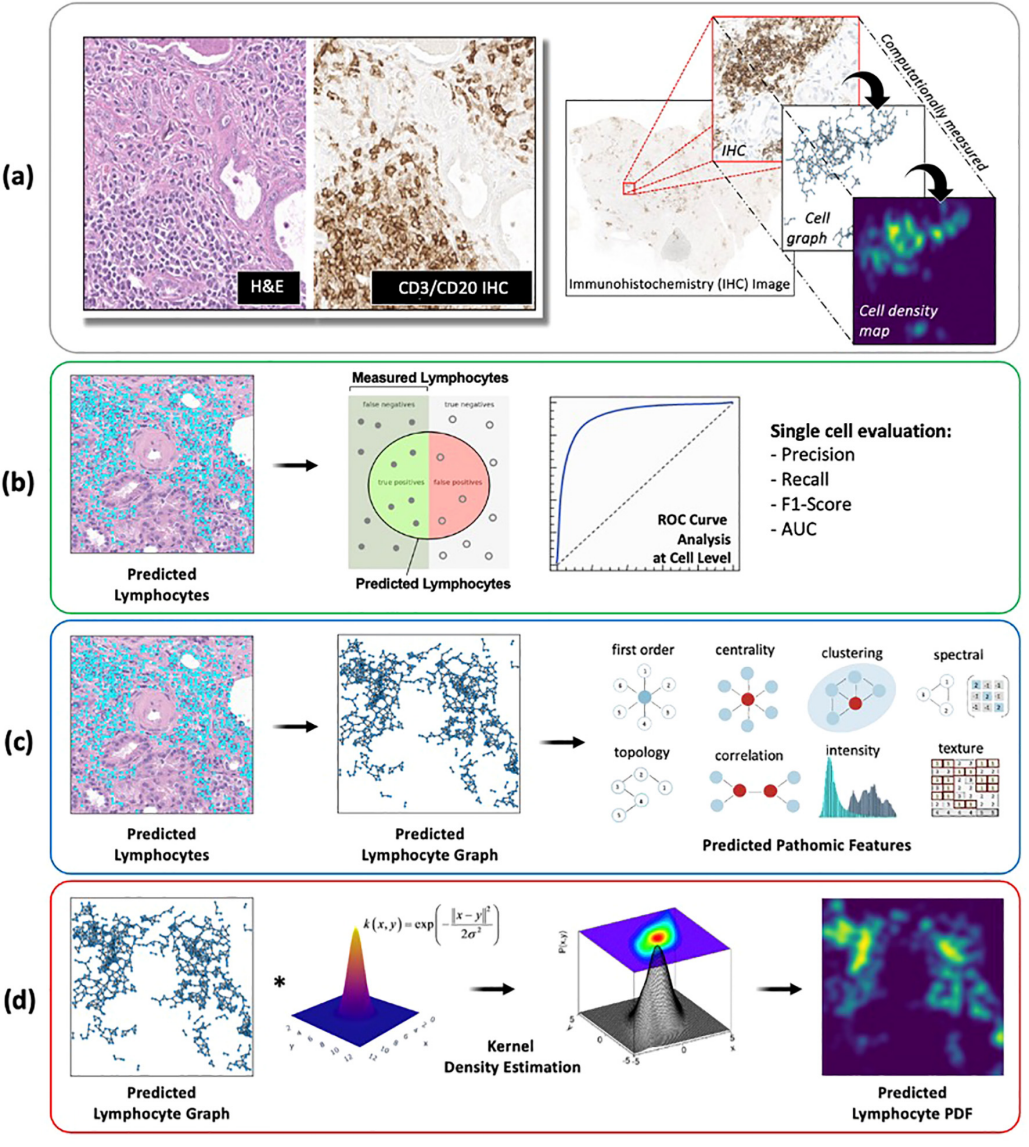
Deep learning model evaluation metrics at different length-scales. **(A)** All deep learning results were compared to IHC-based computational measurements of nuclei counts, nuclei topological graphs, and nuclei density maps. **(B)** Local model performance of individual nuclei predictions was based on analysis of precision-recall curves and receiver operating characteristic (ROC) curves. **(C, D)** Global model performance of pattern-preserving features of the immune microenvironment was based on **(C)** extraction of graph features, where graph nodes represent individual lymphocytes detected via deep learning, and graph edges characterize the spatial connections between different lymphocytes; and **(D)** structural similarity of lymphocyte probability density functions (PDF), estimated based on a kernel density estimation of the predicted lymphocyte graphs using a radial basis function.

Using the internal testing set, model performance was evaluated at two different length-scales relative to IHC measurements (**Figure 6A**): **(1)** a local length-scale to characterize the raw, cell-by-cell deep learning performance of detecting lymphocytes vs. non-lymphocytes; and **(2)** a global length-scale to characterize pattern-preserving pathomic features of the immune microenvironment derived from deep learning detection of lymphocytes. Local model performance was based on analysis of precision-recall curves and receiver operating characteristic (ROC) curves (**Figure 6B**). Global model performance was based on extraction of pathomic graph features and texture features, where graph nodes represent individual lymphocytes detected via the deep learning model, and graph edges characterize the spatial connections between different lymphocytes (**Figure 6C**). Graph features were compared between the *predicted* lymphocytic patterns (based on deep learning detection of lymphocytes as graph nodes) and the *measured* lymphocytic patterns (based on the measured IHC staining of lymphocytes as graph nodes) via arctangent percent error (17). To calculate texture features, a radial basis function was convolved with the graphs to estimate the lymphocytic probability density function (PDF) (**Figure 6D**). From the *predicted* lymphocytic PDF on H&E, texture features were extracted and compared to the *measured* lymphocytic PDF on IHC. Finally, the structural similarity index measure (SSIM) (18) was calculated between the *predicted* and *measured* PDFs as an overall description of predicted topological fidelity. Texture feature definitions are adopted from the Image Biomarker Standardization Initiative (IBSI) (19) and graph feature definitions are listed in **Table 2**.

**Table 2:** Feature definition and explanation for graph features.

Feature Name	Explanation
Centrality_PageRank	Measuring the importance of a node by other important nodes connected to it
Centrality_NodeBetweenness	Measuring the centrality of a node using the number of shortest paths that pass through this node
Centrality_EdgeBetweenness	Measuring the centrality of an edge using the number of shortest paths that pass through this edge
Centrality_CentralPointDomiance	Calculating the central point dominance given the betweenness of each node
Topology_MaxCardinality	Finding the maximum subset of a graph such that no two edges share a common node.
NodeDegree	Node degree for each node
EdgeDistance	Distance between each pair of connected nodes
Spectral_Laplacian	Laplacian matrix for this
Correlation_Assortativity	Measuring how nodes with different types tend to connect with each other
Correlation_ScalarAssortativity	Measuring how nodes with similar degrees tend to connect with each other
Clustering_Global_Coefficient	Computing clustering score of a graph using the ratio of number of triangles and number of connected triples
Clustering_TriangleCount	Number of triangles in a graph
Clustering_TripleCount	Number of connected triples in a graph

### 2.6 Independent testing of lymphocyte deep learning model on an external dataset of diverse human tissues

To independently evaluate the generalization capacity of the developed lymphocyte deep learning model, we applied it to a publicly-available, external dataset. Specifically, we analyzed testing data from the previously completed *Multi-organ Nuclei Segmentation and Classification* (MoNuSAC) Grand Challenge (20), which is designed to systematically evaluate deep learning detection and classification of different cell types on digital pathology. Since MoNuSAC data consists of four different human tissues (breast, kidney, lung, and prostate), it allows us to explore whether our lymphocyte model performs consistently across different human tissues and diverse biological contexts. In total, we applied our model to 146 H&E regions-of-interest (ROI) from 46 TCGA patients, where manual annotation of lymphocytes is available as ground-truth labels as part of the MoNuSAC Grand Challenge. On inference, we calculated precision, recall, and F1 score, as well as Panoptic Quality (PQ), which was the evaluation metric used in the MoNuSAC Grand Challenge.

### 2.7 Experimental confirmation of lymphocyte deep learning model in a genetically engineered mouse model

**Figure 7:**
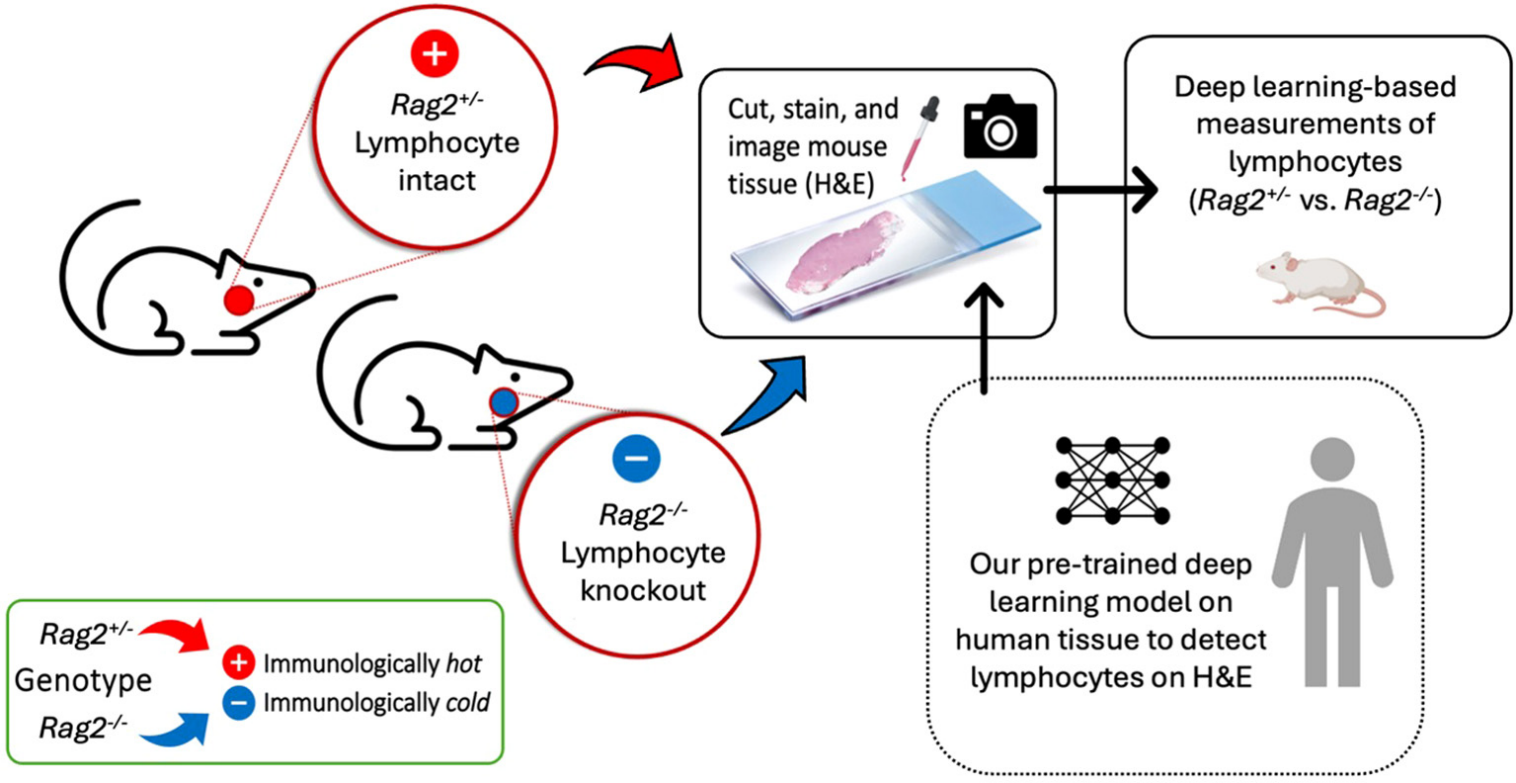
Experimental confirmation of lymphocytic identification by deep learning in a genetically engineered mouse model. A preclinical experiment was performed using mice that have been genetically engineered to lack mature lymphocytes as a negative control. Spleens and thymuses from *Rag2^-/-^* or littermate control *Rag2^+/-^* mice were formalin-fixed, paraffin-embedded, cut, stained with H&E, and imaged on a whole slide digital pathology scanner. A pre-trained deep learning algorithm, independently developed on human tissue to detect lymphocytes on H&E images, was applied to the mouse tissue to measure differences in lymphocytic infiltrate between *Rag2^-/-^* and *Rag2^+/-^* mice. Homozygous knockout mice (*Rag2^-/-^*) were used as negative controls because they lack mature lymphocytes. A heterozygous mouse (*Rag2^+/-^*) with one intact allele was used as a positive control.

To experimentally confirm our lymphocyte model, we conducted a preclinical experiment (**Figure 7**) on mice genetically engineered to lack mature lymphocytes as a negative control. Here, the immune microenvironment of the mice was systematically modulated under experimental conditions (i.e., by genotype) to verify that our deep learning model accurately and reliably identifies lymphocytes. Essentially, we asked the simple question, “what would happen if a deep learning algorithm designed to detect lymphocytes experiences an organism genetically engineered not to have lymphocytes?” To address this question, we evaluated spleens and thymuses from mice genetically engineered to lack *Rag2* (i.e., a gene required for lymphocyte maturation). Homozygous knockout mice (*Rag2^-/-^*) were used as a negative control because they do not produce mature lymphocytes and thus lack the biological basis of the deep learning positive class label. Meanwhile, a heterozygous littermate mouse with one intact allele (*Rag2^+/-^*) was used as a positive control.

#### 2.7.1 Animal model

All animal studies were performed in accordance with protocols approved by the Duke University Institutional Animal Care and Use Committee (IACUC) and adhered to the NIH Guide for the Care and Use of Laboratory Animals. *Rag2^+/-^* and *Rag2^-/-^* mice were bred at Duke University. The *Rag2* gene is essential for B and T cell maturation. Homozygous knockout mice (*Rag2^-/-^*) were used as negative controls because they do not contain mature lymphocytes. A heterozygous mouse with one intact *Rag2* allele (*Rag2^+/-^*) was used as a positive control with mature lymphocytes present.

#### 2.7.2 Experimental measurements of lymphocytes in genetically engineered mice

Mice were euthanized via CO_2_ inhalation. Their spleen and thymus were excised, formalin-fixed, and paraffin-embedded. Tissue sections were cut at 2 microns, stained with H&E, and scanned on the same Leica Aperio AT2 imaging system utilized in Section 2.1 above. Our pre-trained deep learning model, developed on human data per Section 2.4 above, was directly applied to the H&E images to calculate lymphocytic density. Lymphocytic density measurements were compared between *Rag2^+/-^* and *Rag2^-/-^* mice. Importantly, the deep learning model was directly applied to this experimental data without any re-training, fine-tuning, or transfer learning. Therefore, the mouse tissues serve as a final testing set for the deep learning model, where the biology is systematically controlled (i.e., by mouse genotype) to evaluate the performance of this model under defined experimental conditions.

## 3 Results

### 3.1 Image registration of H&E and IHC at single-cell resolution and corresponding lymphocyte training labels

We generated a total of 320 matched pairs of H&E/IHC tiles from 18 patient cases, evenly distributed among minimal, sparse, and dense lymphocytic inflammation. Following image registration of H&E to IHC, the average normalized cross correlation coefficient was 0.89 ± 06, suggesting minimal error from shift artifacts. In total, 111,110 nuclei were automatically segmented on the H&E via StarDist. Of the segmented nuclei, 45,611 nuclei were labelled as lymphocytes based on the co-registered CD3/CD20 IHC reference.

### 3.2 Evaluation of lymphocyte deep learning model on internal testing data

**Figure 8:**
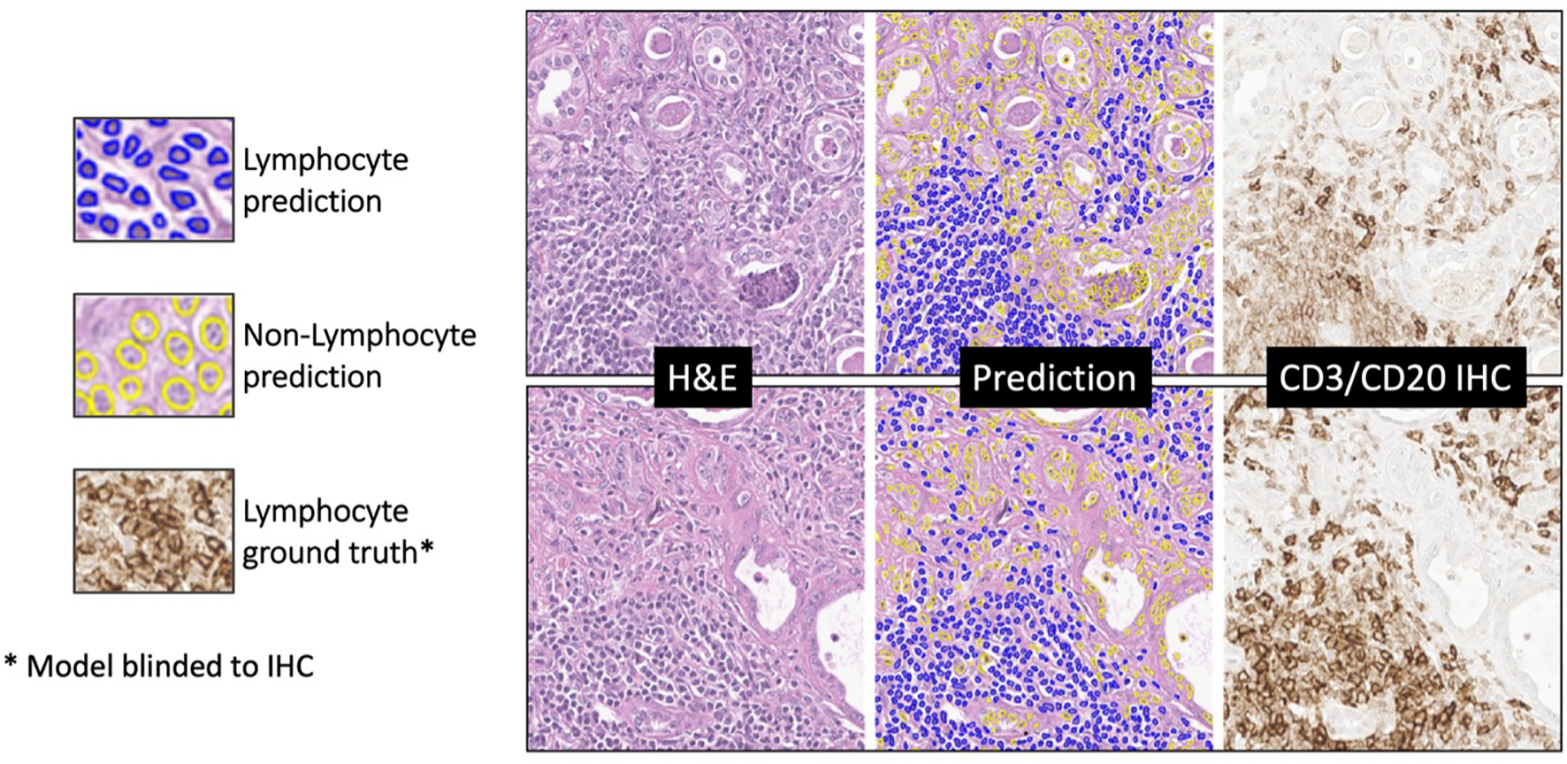
Deep learning lymphocyte prediction examples. From left to right are input H&E patch (1024x1024), model prediction, and CD3/CD20 IHC reference. Predicted lymphocytes are highlighted in blue, while other cells are in yellow. Notably, the model’s predictions closely resemble the brown regions in the IHC reference, indicating its ability to accurately identify lymphocytes in H&E-stained histopathology images.

**Figure 9:**
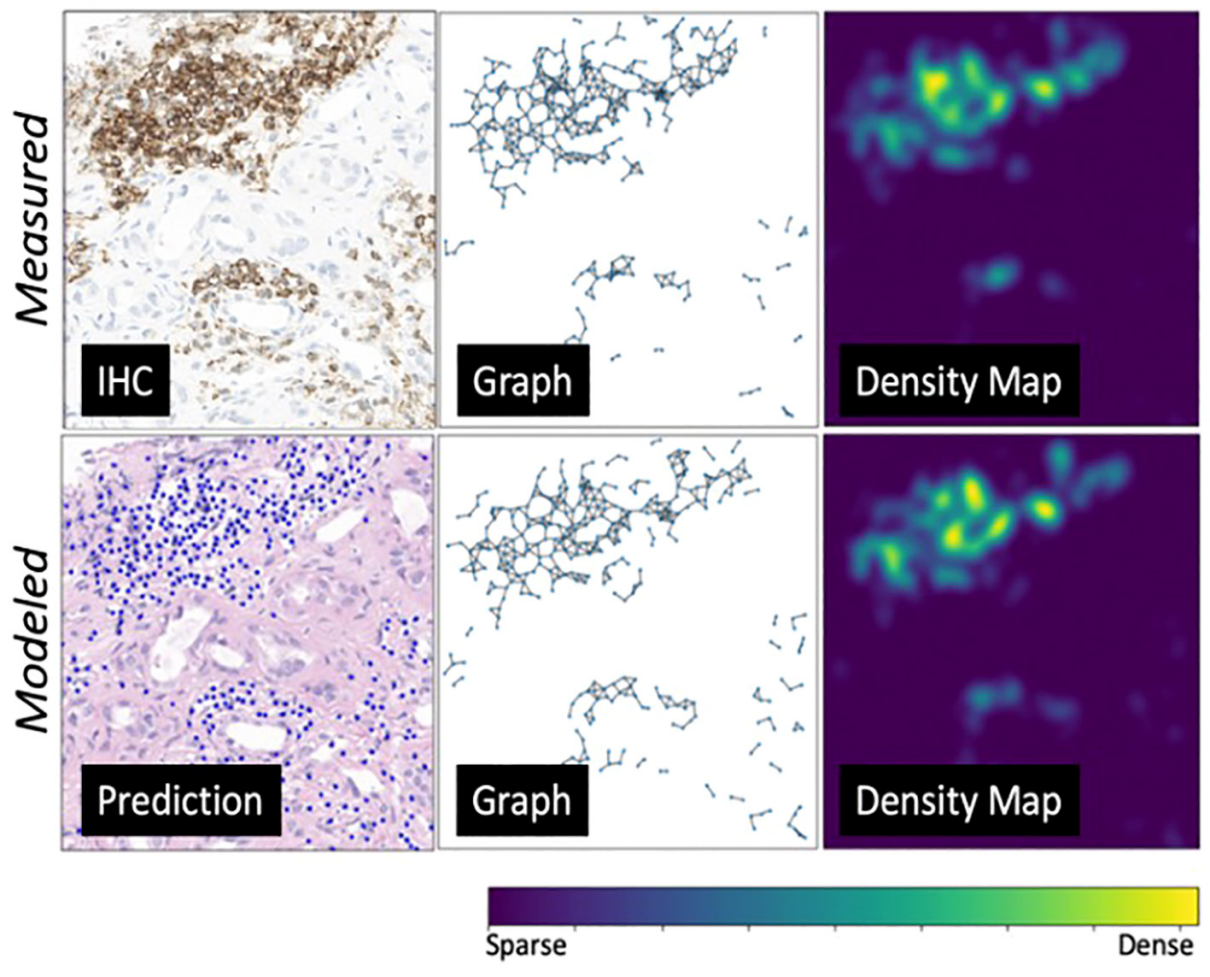
An environmental-level modeling system example. From left to right are predicted lymphocytes, cell graphs showing spatial relationship among lymphocytes, and cell probability density maps (transformed from the cell graphs). The top row showcases the ground truth measurements, while the bottom row presents the modeled measurements. This figure illustrates a strong alignment between the ground truth and prediction model.

On the internal testing set, model precision, recall, and f1 score were 0.74 ± 11, 0.73 ± 10, and 0.73 ± 11, respectively, with an area under the curve (AUC) of 0.78 ± 15. **Figure 8** shows illustrating examples of lymphocyte predictions on H&E testing data relative to corresponding ground-truth CD3/CD20 IHC images. These results demonstrate a strong concordance between the predicted lymphocytes (i.e., the computationally derived blue signal) and the shift-invariant ground-truth CD3/CD20 IHC (i.e., the measured brown signal). The average time to process a 1,024×1,024 image tile was 18 seconds on a single RTX A6000 with 48 GB RAM. As illustrated in **Figure 9**, the average structural similarity between the *predicted* lymphocytic PDFs and the *measured* lymphocytic PDFs was 0.86 ± 06. We found that 19.5% of pathomic features demonstrated a mean error of <10%, and 56.8% of features demonstrated a mean error of >30%. The calculated errors of all pathomic graph features and texture features are reported in the **Supplementary Material**.

On feature extraction from the *predicted* lymphocytic graphs vs. the *measured* lymphocytic graphs, we found that the graph features with the lowest errors were: *First Order Edge Distance Mean* (3.3%), *Laplace Matrix Spectrum Standard Deviation* (10.1%), *Topology Max Cardinality Matching Probability* (11.1%), *Topology Max Cardinality Not Matching Probability* (11.7%), and *First Order Vertex Degree Mean* (15.3%). Meanwhile, the graph features demonstrating the highest errors were *Betweenness of Vertex Mean* (75.1%), *Betweenness of Vertex Standard Deviation* (73.2%), *Betweenness of Edge Standard Deviation* (71.1%), *Central Point Dominance* (70.4%), and *Betweenness of Edge Mean* (60.0%).

On feature extraction from the *predicted* lymphocytic PDFs vs. the *measured* lymphocytic PDFs, we found that the texture features with the lowest errors were *Gray Level Co-occurrence Matrix Inverse Difference* (0.1%), *Gray Level Dependence Matrix Large Dependence Emphasis* (0.2%), *Gray Level Co-occurrence Matrix Correlation* (7.1%), *Gray Level Co-occurrence Matrix Maximal Correlation Coefficient* (7.1%), and *Gray Level Co-occurrence Matrix Information Measure of Correlation 1* (13.3%). Meanwhile, the texture features demonstrating the highest errors were *Gray Level Co-occurrence Matrix Cluster Prominence* (71.3%), *Gray Level Co-occurrence Matrix Cluster Shade* (65.5%), *Neighborhood Gray Tone Difference Matrix Complexity* (59.9%), *Neighborhood Gray Tone Difference Matrix Contrast* (54.3%), and *First Order Robust Mean Absolute Deviation* (32.4%).

### 3.3 Independent testing of lymphocyte deep learning model on an external dataset of diverse human tissues

**Figure 10:**
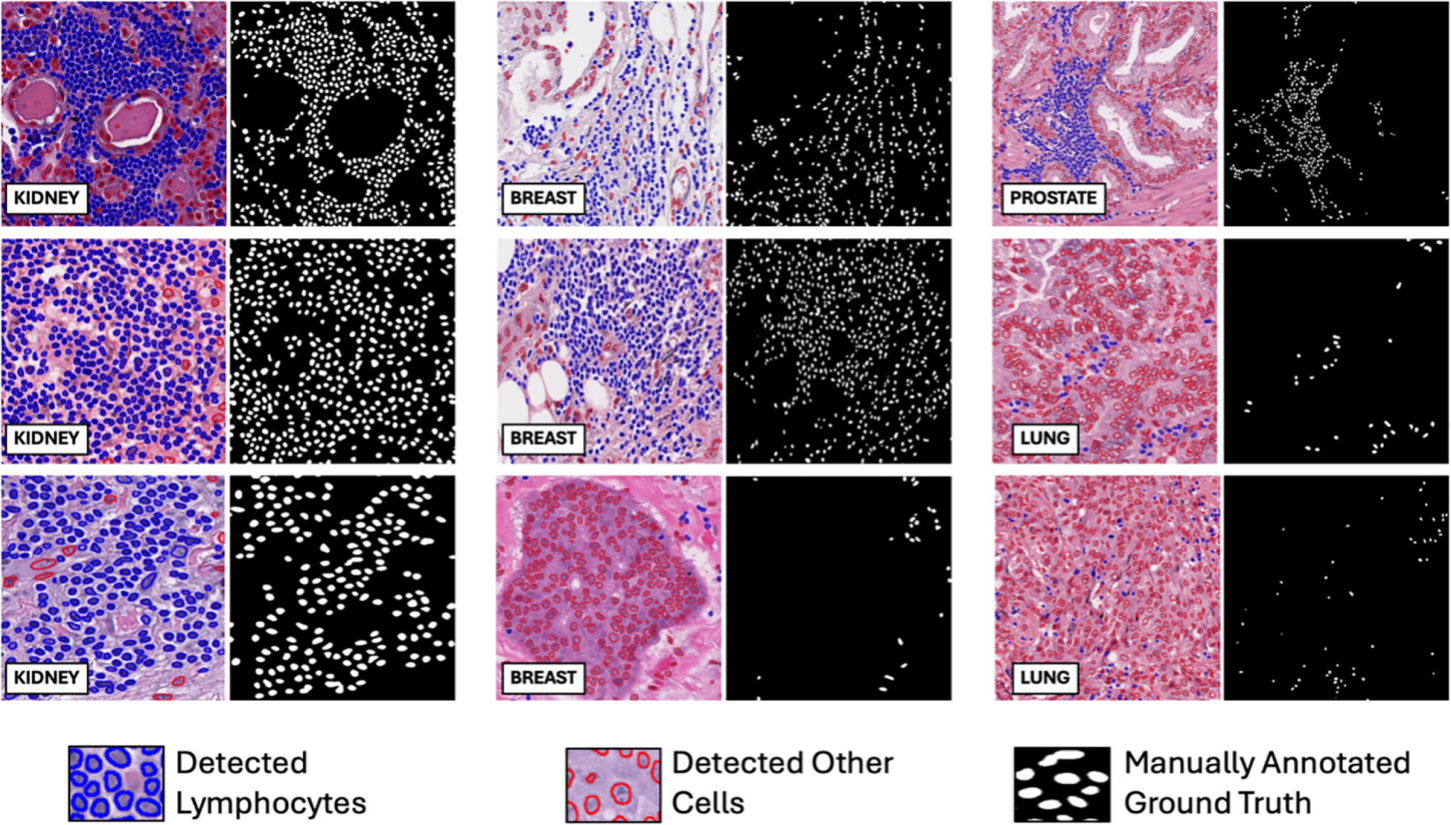
Illustrating examples of lymphocyte detection via deep learning on an external dataset of diverse human tissues. The external MoNuSAC dataset consists of four different human tissues (breast, kidney, lung, and prostate). Each ROI in the figure is paired with a ground truth lymphocyte mask (binary mask, where white represents ground truth lymphocytes) on the right. Blue and red contours represent model-detected lymphocytes and other cells, respectively.

On the external human testing set, model precision, recall, and f1 score were 0.45 ± 33, 0.87 ± 24, and 0.53 ± 31, respectively, with an AUC of 0.71 ± 0.15. A summary of external testing results, including individual performance metrics of specific tissue types, are reported in **Table 3**. The illustrating examples shown in **Figure 10** demonstrate that the model was able to generalize well across diverse human tissues and different biological contexts. Finally, the model achieved a PQ score of 0.43, which is consistent with the published MoNuSAC Grand Challenge leaderboard statistics (average PQ = 0.39 ± 13; range of PQ = [0.10, 0.56]; N=13).

**Table 3:** External validation of lymphocyte detection on diverse human tissues from the public MoNuSAC dataset.

	ROI count	Cell count	Precision	Recall	F1	AUC	PQ
Breast	43	4210	0.48±0.38	0.73±0.34	0.52±0.35	0.73±0.20	0.40±0.27
Kidney	30	3628	0.49±0.33	0.93±0.14	0.58±0.31	0.68±0.14	0.46±0.24
Lung	34	2828	0.41±0.27	0.90±0.15	0.52±0.26	0.73±0.12	0.43±0.21
Prostate	39	3315	0.42±0.30	0.96±0.12	0.52±0.30	0.70±0.12	0.42±0.24
Overall	146	13981	0.45±0.33	0.87±0.24	0.53±0.31	0.71±0.15	0.43±0.24

### 3.4 Experimental confirmation of lymphocyte deep learning model in a genetically engineered mouse model

**Figure 11:**
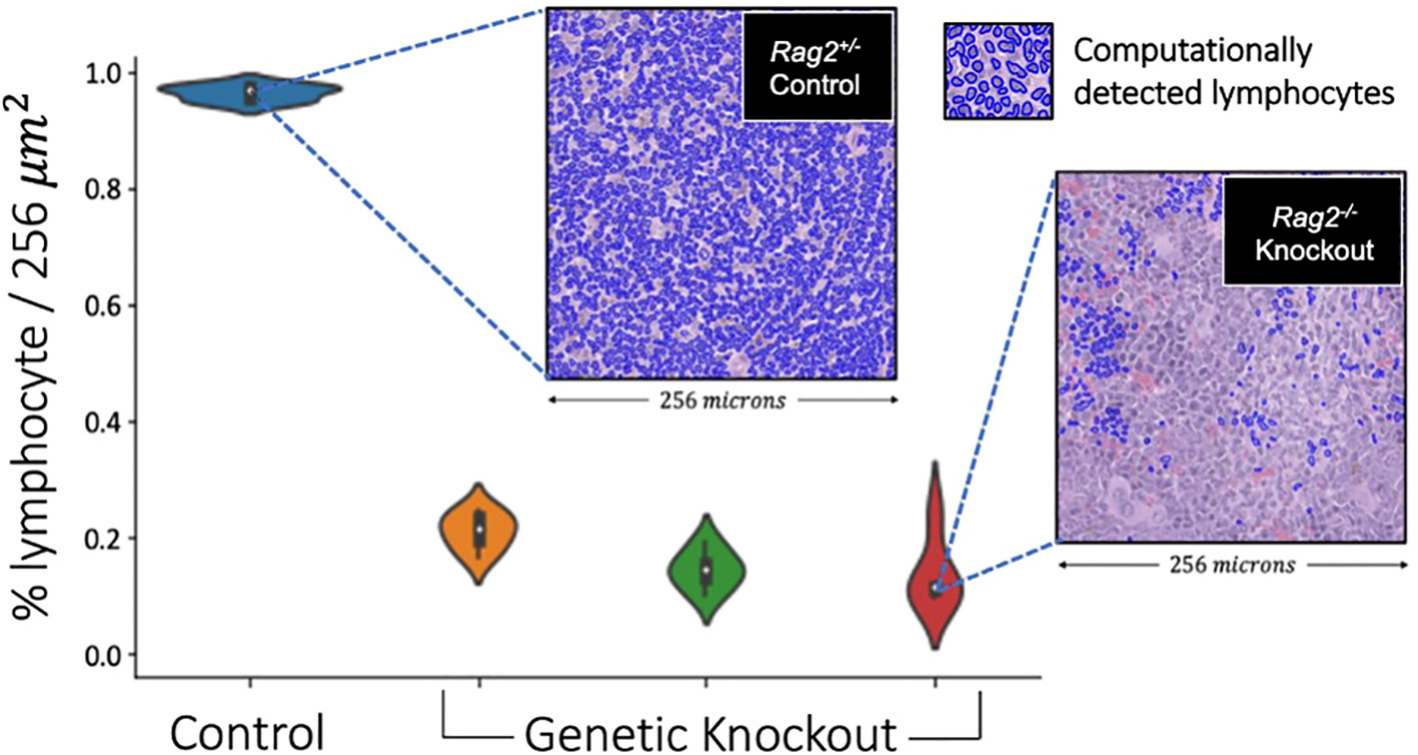
Validation of lymphocyte identification by a deep learning model in a genetically engineered mouse model. The violin plots depict the distribution of lymphocyte percentage in the spleens across 256x256 image patches of different mice. The *Rag2^-/-^* genetic knockout mice demonstrated an average lymphocyte density of 16.2%, which was significantly smaller compared to the *Rag2^+/-^* experimental control mouse of >90% (p<0.0001, ANOVA). These experimental data suggest that our deep learning model generalized across species and tissue types, capturing at least the basic aspects of lymphocytic immune response.

**Figure 12:**
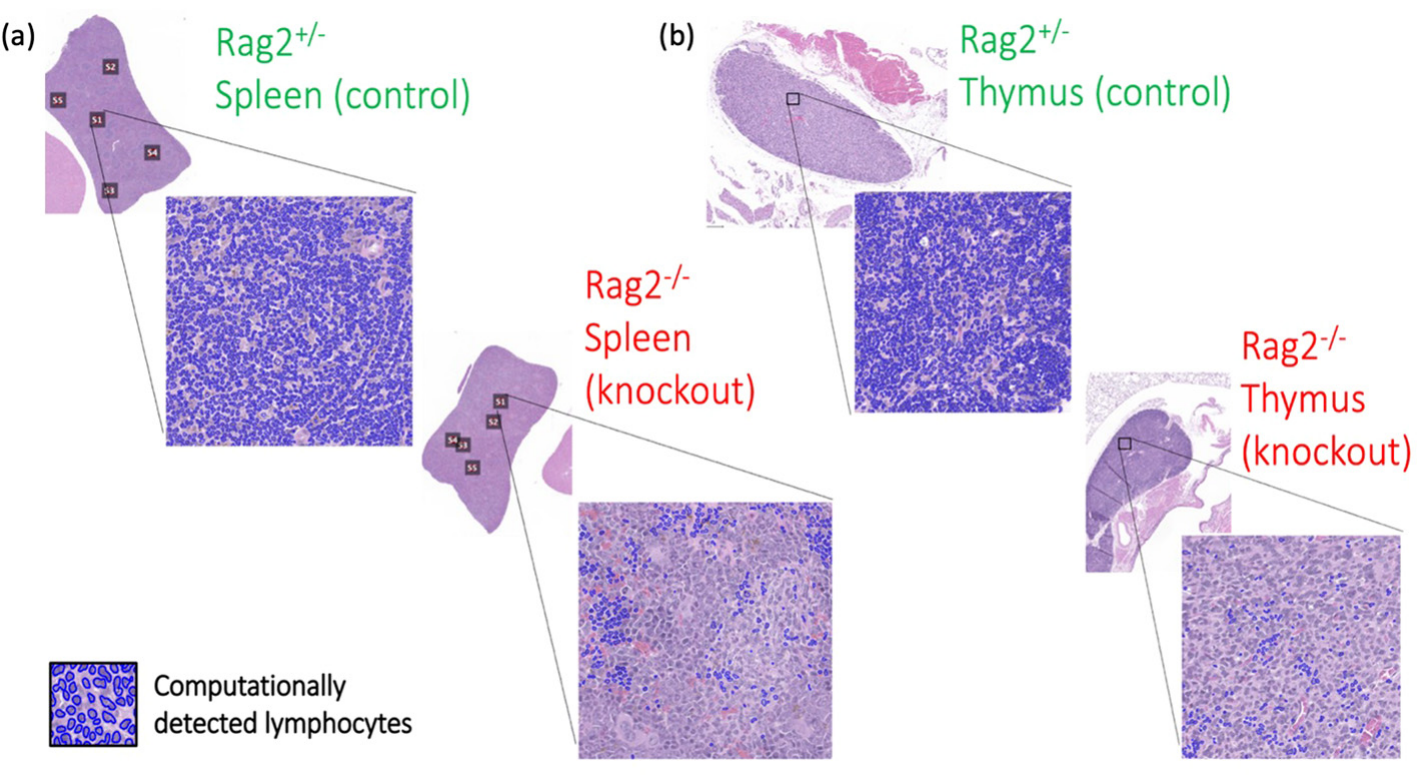
Differences in tissue immune phenotype in genetically engineered mice. Computationally-detected lymphocytes via deep learning inference are shown in blue for both the **(A)** spleen and **(B)** thymus. Qualitative differences in lymphocytic architecture were identified by deep learning in the *Rag2^-/-^* genetic knockout mice compared to the *Rag2^+/-^* experimental control.

On pre-clinical interrogation of our deep learning model, we were able to reliably measure lymphocytes in lymphoid tissues from genetically engineered mice. Experimental results are shown in **Figure 11**, where an average lymphocyte density of 96.5 ± %1% was measured in the *Rag2^+/-^* (i.e., lymphocyte-intact) genotype compared to 16.2 ± %5% in the *Rag2^-/-^* (i.e., lymphocyte knockout) genotype (p<0.0001, ANOVA). Qualitative interpretation showed distinct differences in lymphocyte density on H&E when comparing the spleen (**Figure 12A**) and thymus (**Figure 12B**) in *Rag2^+/-^* versus *Rag2^-/-^* mice.

## 4 Discussion

This study introduced an integrated research design to characterize lymphocytic infiltration on H&E WSIs across different tissue types and species. Our approach extends the capability of lymphocyte quantification to archival digital images of H&E-stained tissue without requiring IHC. Furthermore, it leverages powerful computational analysis tools to capture spatial characteristics of lymphocytic inflammation in tissues. By combining computational image analysis with novel tissue processing procedures (matched H&E and IHC on the same slide) and genetically engineered mouse models, we demonstrated a rigorous approach to deep learning algorithm development and evaluation under well-controlled laboratory conditions. Although image-based characterization of lymphocytes identified by IHC staining has shown success in various cancer studies, our approach extends this capability to digitized H&E-stained slides, overcoming limitations associated with the cost and feasibility of IHC in routine pathology and retrospective research. Furthermore, deep learning algorithms that identify different cell types on H&E WSIs offer the opportunity to simultaneously analyze the topology of those cells and to capture crucial structural details of the tissue, enabling examination of a broader spectrum of cell types and tissue organization.

A key innovation of our approach is the tissue processing and image registration procedure to generate efficient class labeling on H&E images with high accuracy and precision. This in turn enables robust deep learning model development. In biomedical imaging applications of deep learning, generation of reliable ground-truth labels remains a major challenge (21, 22). To help address this issue, we optimized training label fidelity through image registration of H&E and IHC staining performed on the same slide at single-cell resolution, achieving pixel-perfect, ground-truth labels. As the knowledge base of our model is IHC antibodies with high specificity for lymphocytes – and not manual annotation by pathologists – the training data do not suffer from intra- or inter-observer variability.

Furthermore, our approach is fundamentally different than clinical pathology workflows and other studies that typically employ H&E and IHC staining on sequential slides cut from a tissue block. While this approach generates both stains on the same specimen, the information content is not shift-invariant, making it non-trivial for deep learning applications that resolve data at the length-scale of individual immune cells. Unlike staining sequential slides, performing H&E and IHC staining on the same tissue enables data curation at single-cell resolution and is thus well-suited for deep learning applications at the physical length-scales of individual cells.

Same-tissue processing also enables error propagation of downstream pathomic features derived from the ensemble of detected cells. This is important because the spatial interplay of cells, as captured by representative pathomic features, is essential to spatially characterizing tissue microenvironments. For example, spatial cell-to-cell interplay has been shown to be linked with therapeutic responses and tumor biology (23–25), as well as broader applications from intracellular to extracellular conditions and associations with various tissue microenvironments (26–29). Many of these topological characteristics can be computationally measured in various ways, including cell geographic clusters, cell density distributions (30), cell topological graphs (31), cell clouds (32), and graph neural networks (GNNs) (33), potentially leading to deeper insights into the mechanisms at play in different pathological and immunological states.

Our experiment comparing *predicted* pathomic features on H&E to *measured* pathomic features on IHC suggest that the error-rate of pathomic extraction is largely feature-specific. That is, certain pathomic features are more sensitive than others to the same errors in the initial deep learning inference. This has major implications on pathomic biomarker models that rely on an upstream deep learning framework for initial cell detection. For example, variation induced by deep learning errors, which leads to inconsistencies in pathomic feature extraction, may consequently result in variability in the computational biomarkers derived from these features. Same-tissue staining at single-cell resolution enables characterization of this effect, such that propagation of error for individual features can be better understood. This may be a useful feature selection strategy to remove highly variable features that would otherwise introduce noise into downstream biomarker models. Our results demonstrate that even with modest deep learning performance, there are pathomic features which still preserve the global topological patterns of the immune microenvironment. To the best of our knowledge, there is limited prior research studying such pathomic feature propagation of error.

Our external testing results on diverse human tissues were similar to the published MoNuSAC Grand Challenge results (PQ = 0.43 vs. PQ = 0.39 ± 13), implying that the performance of our model is comparable to other lymphocyte deep learning models in the literature. Our lymphocyte model thus demonstrated reasonable generalization when applied to an independent testing dataset of different human tissues, suggesting that it can perform consistently across diverse biological contexts. Furthermore, these external data were of variable stain quality, tissue processing, and image acquisition, which is important because laboratory conditions cannot always be easily replicated across institutions.

However, there are several factors that may contribute to differences in model performance between the internal and external datasets. First, the manual lymphocyte annotation of the MoNuSAC Grand Challenge dataset is fundamentally different than the IHC-based measurements of our internal dataset, potentially leading to imprecise definitions of ground-truth. Second, the diverse tissue types of the MoNuSAC Grand Challenge data – which were not observed in our internal data – may contribute to a biological domain shift that requires fine-tuning of models to specific pathologies. Our external testing results support this concept, where high recall scores indicate stable lymphocyte morphology detection in different tissue conditions, yet lower precision scores suggest variation in tissue content not present in training data, where objects with similar characteristics as lymphocytes were mis-recognized by the model.

Finally, the major novelty of our paper was the cross-species validation of our lymphocyte model, where we trained our model on human tissues, and externally validated it using a genetically engineering mouse model. This is significant, because while deep learning models demonstrate remarkable proficiency in pattern recognition, their limitations in mechanistic understanding are evident. Often, these models fall short in capturing fundamental biological characteristics beyond surface-level image representation. This gap underscores a critical challenge, where purely data driven solutions alone may not sufficiently elucidate the intricate mechanisms underlying immune responses. Consequently, there is a pressing need for complementary experimental testing to validate and refine these models under controlled conditions. We also note that data assimilation methods (i.e., where mechanistic models from either physics or biology are integrated with data-driven solutions, such as deep learning (34–36)), may provide a more rigorous description of the imaging phenotype and a better understanding of deep learning (37, 38). Our mouse model results suggest that integrating computationally-derived spatial analysis with traditional basic science approaches could enhance our understanding of complex biological systems.

Although our research demonstrates several technological innovations and novel findings, our study is not without limitations. First, our tissue processing scheme may result in tissue deformations during the re-staining procedure, which could result in errors during image registration. However, these deformations are minimal when compared to those from cutting the tissue. Because we only cut the tissue once, our rigid registration errors should be smaller than with sequential cuts, where deformable image registration is required. Second, while total cell counts were substantial in our data, case level variability was limited to only 18 unique patient samples used in model development. This is unfortunately a consequence of our prospective data curation and intricate tissue processing procedure, which emphasized data quality over data quantity. We partially addressed this limitation by employing case level partitioning for training, validation, and internal testing, as well as independent external testing on both human and murine datasets. Future work needs to apply these strategies to larger, more diverse data sets. Third, the current paper only focuses on lymphocytes, but immune responses are more diverse and include non-lymphocytic pathologies (e.g., neutrophils, macrophages, etc.). Since our proposed tissue processing pipeline can be generalized to other antibodies, future work should focus on development of additional models for different immune cell types. This would enable a more comprehensive analysis of the immune microenvironment.

In summary, our results demonstrate that deep learning can reliably identify lymphocytes on H&E slides and capture differences in lymphocyte spatial architecture. We plan to continue mechanistic interrogation of this technique in preclinical animal models to obtain a deeper, more holistic understanding of underlying processes that drive disease. This approach will help to ensure that computational predictions are biologically relevant and scientifically robust with the goal of ultimately developing clinically actionable biomarkers.

## Data Availability

The raw data supporting the conclusions of this article will be made available by the authors, without undue reservation.
