# Supplementary material for: Computational staining of CD3/CD20 positive lymphocytes in human tissues with experimental confirmation in a genetically engineered mouse model
Source: Front Immunol. 2024 Oct 28;15:1451261. doi: 10.3389/fimmu.2024.1451261 (PMC11550988; doi:10.3389/fimmu.2024.1451261)
Supplement: Supplementary file 1 [file DataSheet1.pdf]

Supplemental Material

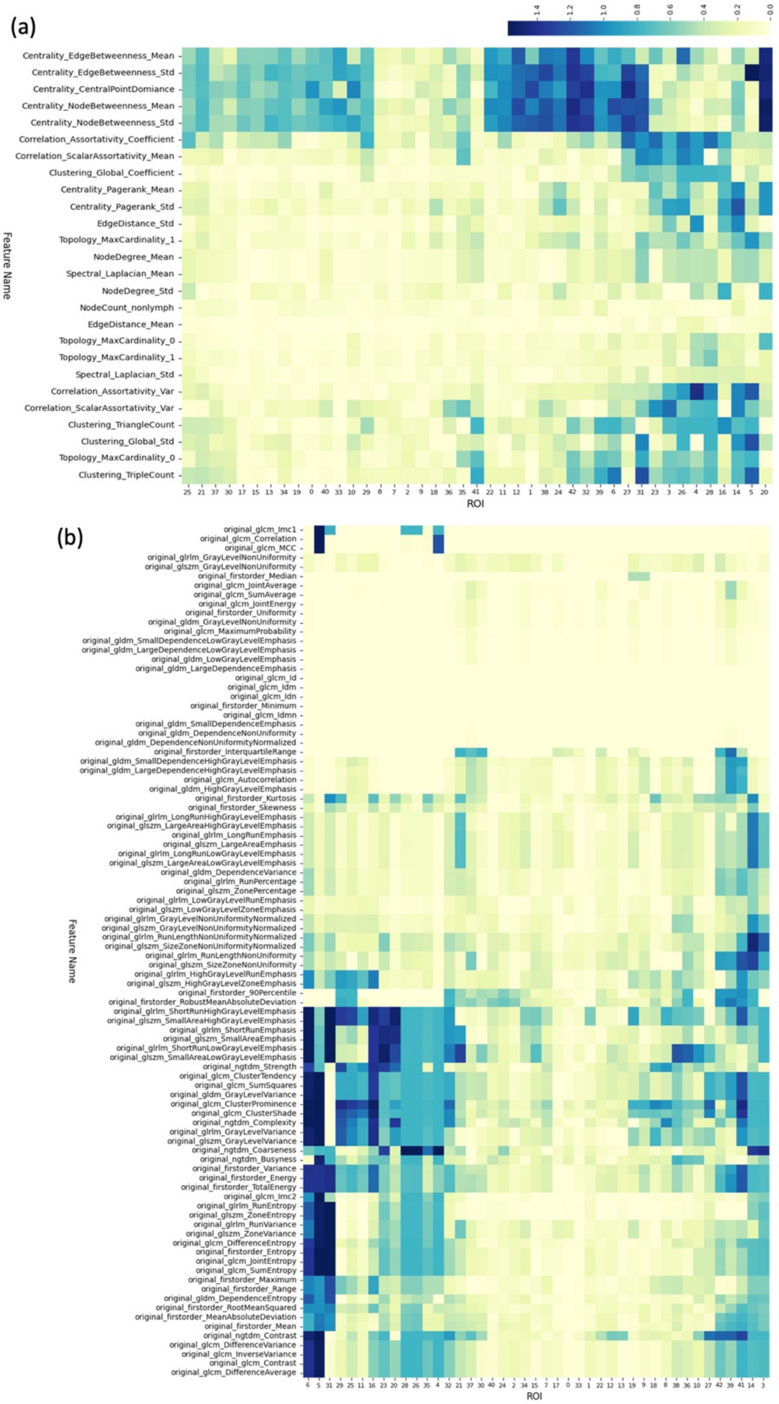

Supplemental Figure 1. Arctangent percentage error (scaling from 0 to 1.5) for each pathomic feature across all testing ROI tiles. (a) Graph features (K=27). Features from First Order and Topology families were more stable than Correlation, Centrality, and Cluster families. (b) Texture features (K=93). Features from Gray Level Co-occurrence Matrices and Gray Level Difference Matrices are generally more stable than those derived from Gray Level Run Length Matrices, Gray Level Size Zone Matrices, or Neighborhood Gray Tone Difference Matrices.
